# Oncometabolite D-2-Hydroxyglurate Directly Induces Epithelial-Mesenchymal Transition and is Associated with Distant Metastasis in Colorectal Cancer

**DOI:** 10.1038/srep36289

**Published:** 2016-11-08

**Authors:** Hugh Colvin, Naohiro Nishida, Masamitsu Konno, Naotsugu Haraguchi, Hidekazu Takahashi, Junichi Nishimura, Taishi Hata, Koichi Kawamoto, Ayumu Asai, Kenta Tsunekuni, Jun Koseki, Tsunekazu Mizushima, Taroh Satoh, Yuichiro Doki, Masaki Mori, Hideshi Ishii

**Affiliations:** 1 Department of Gastroenterological Surgery, Osaka University Graduate School of Medicine, Osaka, 565-0871, Japan; 2 Department of Frontier Science for Cancer and Chemotherapy, Osaka University Graduate School of Medicine, Osaka, 565-0871, Japan; 3 Department of Cancer Profiling Discovery, Osaka University Graduate School of Medicine, Osaka, 565-0871, Japan; 4 Taiho Pharmaceutical Co., Ltd., Chiyoda-ku, Tokyo, 101-0054, Japan

## Abstract

Deranged metabolism is a hallmark of cancer, playing a significant role in driving the disease process. One such example is the induction of carcinogenesis by the oncometabolite D-2 hydroxyglutarate (D-2HG), which is produced by the mutated enzyme isocitrate dehydrogenase (*IDH*) occurring in subsets of leukaemias and brain tumours. The oncogenic property of D-2HG appears to stem from its ability to interfere with the activities of α-ketoglutarate-dependent dioxygenases, including the Jumonji family histone demethylases. Here, we find in colorectal cancer cells that even in the absence of IDH mutation, the levels of D-2HG and its enantiomer L-2HG were elevated through glutamine anaplerosis. D-2HG, but not L-2HG, increased the trimethylation of histone H3 lysine 4 of the promoter region of *ZEB1*, a master regulator of epithelial-mesenchymal transition (EMT), and increased the expression of the *ZEB1* gene to directly induce EMT in colorectal cancer cells. EMT promotes the ability of cancer cells to invade the local tissue and enter into the bloodstream, leading to distant organ metastasis. D-2HG levels were elevated in colorectal cancer specimens, particularly in those associated with distant metastasis, supporting the observations *in vitro* and implicating the contribution of D-2HG in metastasis, the major cause of death in this disease.

Colorectal cancer is the third most common cancer and the fourth most common cause of cancer-related deaths worldwide, leading to approximately 700,000 deaths each year^1^. The frequency with which the disease presents at an advanced stage^2^, and therefore beyond the scope of curative surgical resection^3^, underlies the high number of deaths and emphasises the need for novel treatment strategies, underpinned by a better understanding of the disease.

Deranged metabolism is a hallmark of cancer, including in colorectal cancer^4^, and can encompass many of its components from the increased cellular uptake of glucose and other nutrients to the shift to anaerobic metabolism of glucose to lactate, allowing the rapidly proliferating malignant cells to meet their energetic and biosynthetic demands^5^. Moreover, we and others have found that deranged metabolism also drives oncogenesis^6^ and disease progression^7^,^8^, highlighting the significant role of cellular metabolism throughout the disease process and as a promising target for treatment.

Specific metabolites have been identified to be the crucial link between metabolism and oncogenesis, providing an insight into the molecular mechanisms behind this phenomenon^9^. Metabolites are the intermediate products of metabolism, and are the fuel, biomaterials and signalling molecules of the cells^6^,^10^. It appears to be the latter property of metabolites that endows them with the ability to alter gene expression in cancer cells, including through control of the epigenetic machinery^6^. One such metabolite is D-2-hydroxyglurate (D-2HG), the concentration of which is very low in healthy individuals, but high in subsets of leukaemias and brain tumours that harbour gain of function mutations of the enzymes isocitrate dehydrogenase 1 or 2 (*IDH1* or *2*)^11^. D-2HG induces leukemogenesis^12^ and malignant transformation of astrocytes^13^, but has no known physiological function, and is widely referred to as an “oncometabolite”. The oncogenic nature of D-2HG appears to stem from its ability to interfere with the activity of various α-ketoglutarate-dependent dioxygenases, including ten eleven translocation 5-methylcytosine hydroxylases (TET) involved in DNA demethylation and Jumonji family histone demethylases^11^,^14^, which can lead to widespread changes of DNA and histone methylation, resulting in the disruption of cellular proliferation and differentiation^15^,^16^.

The revelation that 2HG levels are elevated in breast cancer even with wild-type *IDH*, driven by glutamine anaplerosis (Fig. 1A)^17^,^18^,^19^, highlighted that 2HG levels could be elevated in a wider range of malignancies that do not necessarily harbour mutated *IDH*^20^. Also in the azoxymethane mouse model of intestinal cancer, 2HG levels were elevated in the tumours compared to normal tissues^21^, providing further support that the oncometabolite may be of importance in human colorectal cancer.

In renal cell carcinoma, L-2HG, the opposite enantiomer of D-2HG, is elevated secondary to decreased expression of L-2HG dehydrogenase, the enzyme responsible for the metabolisation of L-2HG^22^. L-2HG is also the product of glutamine anaplerosis, but in contrast to D-2HG, it is produced from the reduction of α-ketoglutarate by lactate dehydrogenase A or malate dehydrogenase (Fig. 1A)^23^. L-2HG is thought also to be an oncometabolite, but it is distinct from D-2HG in its bioactivity, given that it does not cause leukemogenesis or astrocyte transformation^12^,^13^.

Here we explore the role of D- and L-2HG in colorectal cancer, and find the levels of these metabolites to be elevated in colorectal cancer cells. D-2HG, but not L-2HG, directly induced epithelial-mesenchymal transition (EMT) in colorectal cancer cells, which is associated with cancer cells acquiring the ability to invade the surrounding tissues and enter the bloodstream, leading to distant organ metastasis^24^. D-2HG also induced histone modifications associated with increased gene expression at the promoter region of the *ZEB1* gene, which encodes a master regulator of EMT. Furthermore, clinical specimens with higher levels of D-2HG were more likely to be associated with distant organ metastasis, supporting the significant contribution of the metabolite in cancer metastasis.

## Results

### D- and L-2HG Levels are Elevated in Colorectal Cancer Cells and is Dependent on Glutamine Metabolism

**Figure 1:**
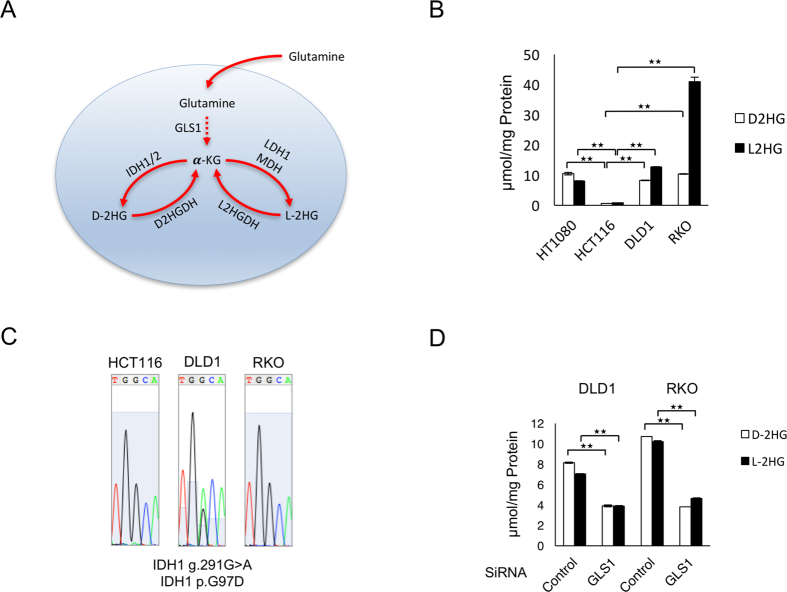
D- and L-2HG levels are elevated in colorectal cancer cells and is dependent on glutamine metabolism. (**A**) The metabolic pathway of the production of D- and L-2HG from glutamine. Glutamine is metabolised to α-ketoglutarate (α-KG) in sequential steps, involving glutaminase (GLS1). α-KG can then be metabolised to D-2HG by IDH1 or 2, or to L-2HG by lactate dehydrogenase 1 (LDH1) or malate dehydrogenase (MDH). Furthermore, D-2HG is converted physiologically to α-KG by D-2HG dehydrogenase (D2HGDH), as is L-2HG to α-KG by L-2HG dehydrogenase (L2HGDH). (**B**) Baseline levels of D- and L-2HG were measured in three colorectal cancer cell lines (DLD1, HCT116, RKO) and a sarcoma cell line HT1080 known to harbour mutant *IDH1* (R132C) by gas chromatography-mass spectrometry (GC-MS). Data are presented as means and standard deviations; **p < 0.01. (**C**) Sanger sequencing confirms the presence of *IDH1* mutation (G97D) in the DLD1 cell line. *IDH1* was wild-type in HCT116 and RKO cell lines. (**D**) D- and L-2HG levels were measured by GC-MS in DLD1 and RKO cell lines, after siRNA interference of GLS1. Data are presented as means and standard deviations; **p < 0.01.

To explore the potential of D- or L-2HG playing a role in colorectal cancer, we began by measuring the levels of the metabolites in colorectal cancer cell lines by gas chromatography-mass spectrometry (GC-MS). In two of the three cell lines analysed (DLD1 and RKO), both D- and L-2HG were elevated at levels comparable to or exceeding the concentrations measured in the sarcoma cell line HT1080, harbouring mutant *IDH1* (Fig. 1B). Given that mutations of *IDH1* and *IDH2* are the widely known cause of elevated levels of D-2HG, these genes were sequenced, confirming the presence of mutated *IDH1* (G97D) in the DLD1 cell line, previously revealed to be present in the same cell line^25^ and to lead to the production D-2HG^26^ (Fig. 1C). However the *IDH1* and *2* enzymes were wild-type in RKO cells, leading to the speculation that D-2HG could be elevated in this cell line instead from glutamine anaplerosis, which would also explain the levels of L-2HG being elevated^18^,^23^ (Fig. 1A). In support of glutamine anaplerosis fuelling the production of D- and L-2HG, the concentration of both metabolites were reduced in the colorectal cancer cells when glutaminase expression was knocked down, an enzyme responsible for the first step of several in the catabolism of glutamine to α-ketoglutarate (Fig. 1D, Supplementary Fig. S1A). Furthermore, the baseline expressions of glutaminase and other enzymes involved in the catabolism of glutamine to α-ketoglutarate, aspartate aminotransferase 2 and glutamate dehydrogenase 1, were elevated in the DLD1 and RKO cell lines relative to the HCT116 cell line (Supplementary Fig. S1B and C). Given gene expression is one of the determinants of metabolic flux, these results suggest that the differences in the expression of enzymes involved in glutamine metabolism may explain the variation in the levels of D- and L-2HG across the colorectal cancer cell lines.

In diffuse large B-cell lymphoma, D-2HG levels have been found to be elevated secondary to mutation of the enzyme D-2HG dehydrogenase (*D2HGDH*)^27^, which has a physiological role of converting D-2HG to α-ketoglutarate. We therefore examined for mutations of *D2HGDH*, and its counterpart for L-2HG, L-2HG dehydrogenase (Fig. 1A) in DLD1, HCT116 and RKO cells through sequencing, and found both enzymes to be wild-type in the three colorectal cancer cell lines. Furthermore, the expression of *D2HGDH* and *L2HGDH* were not consistently lower in DLD1 and RKO cells in comparison to HCT116 cells, as might have been expected if the decreased metabolisation of 2HG was to explain the higher levels of these metabolites in DLD1 and RKO cells (Fig. S1D). These results add further weight to the notion that it is the production of 2HG rather than its metabolisation that governs the levels of D- and L-2HG in colorectal cancer cell lines.

### D-2HG Induces EMT in Colorectal Cancer Cells

**Figure 2:**
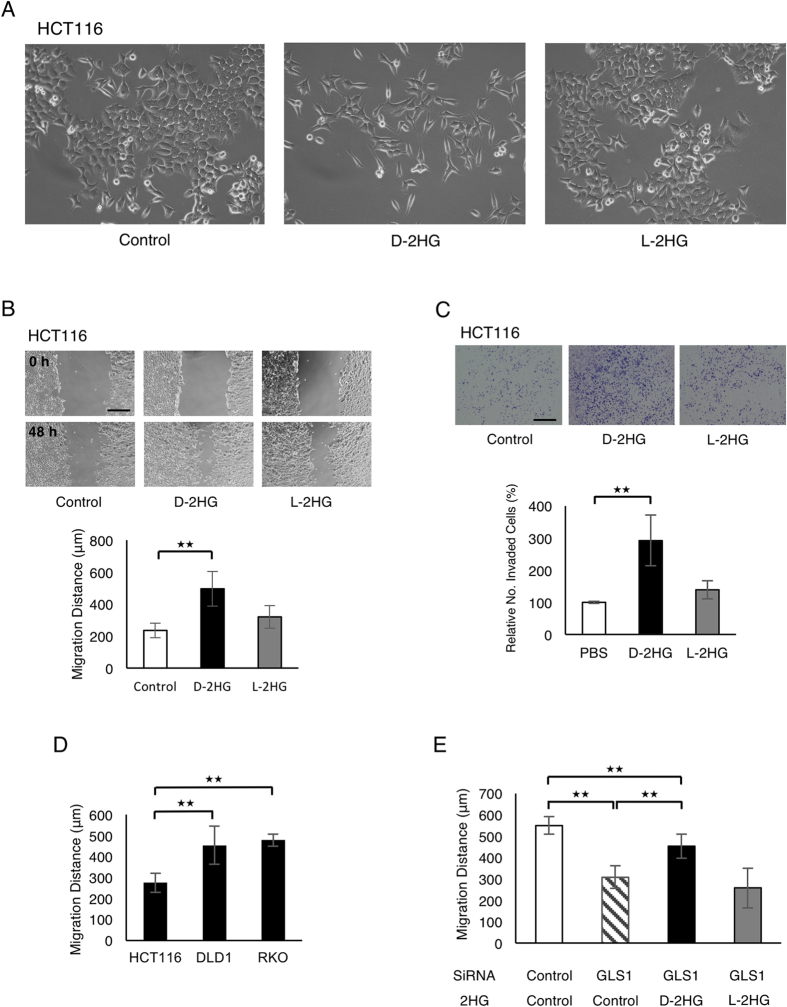
D-2HG is sufficient and necessary to induce a mesenchymal phenotype in colorectal cancer cells. (**A**) The morphology of HCT116 cells are pictured after treatment with D-2HG (250 μM) or L-2HG (250 μM) over 20 passages. (**B**) The migration of HCT116 cells treated with D- or L-2HG (250 μM over 20 passages) was assessed by wound healing assay at 48 hours. Scale bar: 200 μm. (**C**) The invasion of HCT116 cells treated with D- or L-2HG (250 μM over 20 passages) were assessed with the matrigel invasion assay. Scale bar: 400 μm. (**D**) The migration of colorectal cancer cell lines (HCT116, DLD1, RKO) were assessed by wound healing assay after 96 hours. (**E**) The migration of the DLD1 cell line by wound healing assay after siRNA interference of GLS1 and/or treatment with D-2HG octylester. Measurements were taken at 48 hours. Data are presented as means and standard deviations of at least three independent experiments; **p < 0.01.

To examine the potential role of D- and L-2HG in colorectal cancer, these metabolites were administered as octylester derivatives to the HCT116 cell line, in which neither of the metabolites were elevated at baseline. 2HG octylesters are commonly used in place of 2HG given they can permeate across the cell membrane, to be converted by intracellular esterases to 2HG^19^,^22^. After the administration of D-2HG octylester over 20 passages, we found that the colorectal cancer cells transformed into spindle-shaped cells, which can signify the acquisition of the mesenchymal phenotype. In contrast, no obvious changes in cellular morphology were observed with the administration of L-2HG octylester (Fig. 2A). The acquisition of the mesenchymal phenotype would be of clinical significance, since this phenotype is associated with the promotion of the cancer cells’ ability to invade the local tissues and enter the bloodstream, leading to distant organ metastasis^28^. Indeed, HCT116 cells treated with D-2HG octylester acquired increased ability for migration (Fig. 2B) and invasion (Fig. 2C), demonstrating that D-2HG is sufficient to induce some of the cardinal features of the mesenchymal phenotype. Consistent with the above, the basal cellular migration was higher in DLD1 and RKO cells, the cells with elevated D-2HG levels in comparison to HCT116 (Fig. 2D). Cellular migration was inhibited when 2HG levels were reduced through knockdown of glutaminase, but was partially rescued by the administration of D-2HG octylester (Fig. 2E), signifying that cellular migration is also partially dependent on D-2HG.

**Figure 3:**
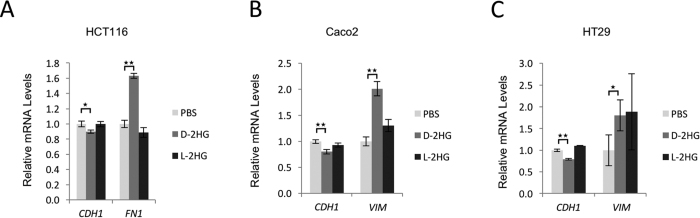
D-2HG induces epithelial-mesenchymal transition in colorectal cancer cells. (**A**) qPCR of epithelial marker (*CDH1*) and mesenchymal marker (*FN1*) in HCT116 cells treated with D- or L-2HG (250 μM) over 20 passages. (**B**) qPCR of epithelial marker (*CDH1*) and mesenchymal marker (*VIM*) in Caco-2 cells treated with D- or L-2HG (250 μM) over 4 passages. (**C**) qPCR of epithelial marker (*CDH1*) and mesenchymal marker (*VIM*) in HT29 cells treated with D- or L-2HG (250 μM) over 2 passages. Data are presented as means and standard deviations of at least three independent experiments; *p < 0.05, **p < 0.01.

The reduced expression of the epithelial marker E-cadherin (*CDH1*), with the concurrent increase in the expression of the mesenchymal markers fibronectin (*FN1*) or vimentin (*VIM*) in the HCT116 cell line, as well as in two other colorectal cancer cell lines Caco2 and HT29 (Fig. 3A–C, Supplementary Fig. S2A and B) confirmed the induction of epithelial-mesenchymal transition by D-2HG across colorectal cancer cells with different genetic background (Supplementary Table S1). These observations were not accompanied by any consistent alteration in the rate of cellular proliferation across the colorectal cancer cell lines treated by 2HG octylesters (Supplementary Fig. S2C).

### D-2HG Directly Induces EMT in Colorectal Cancer Cells

**Figure 4:**
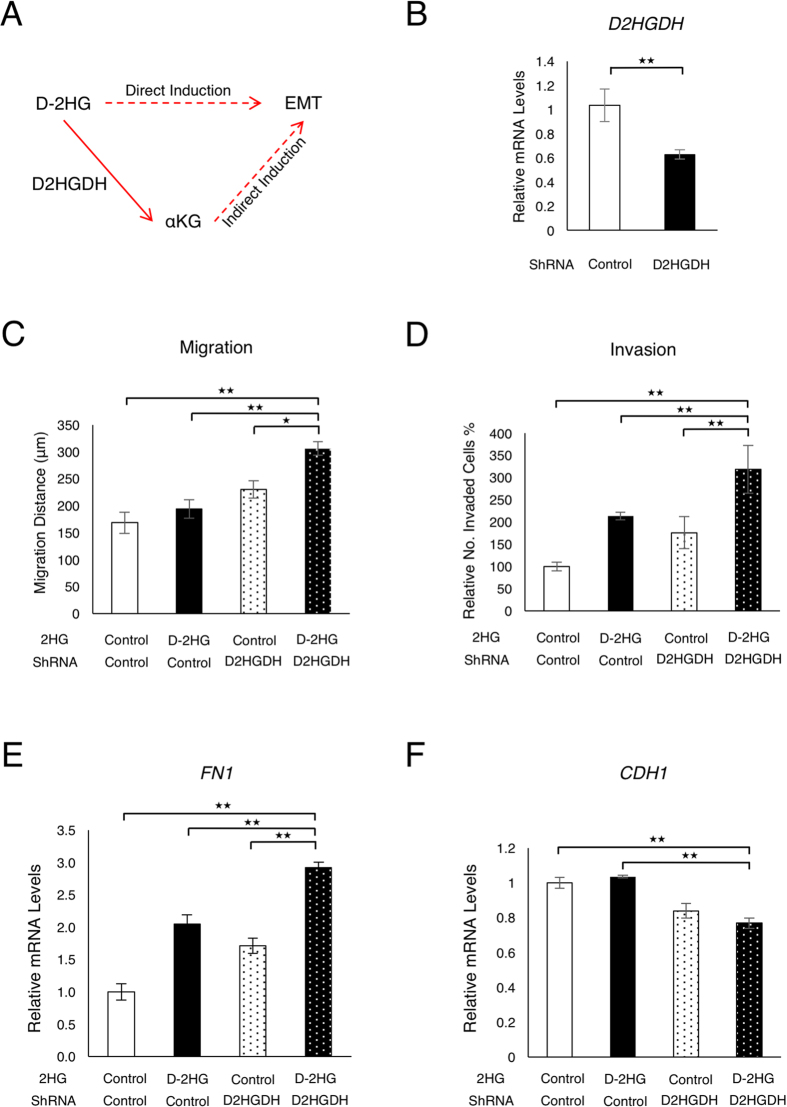
D-2HG is a direct inducer of EMT in colorectal cancer cells. (**A**) D-2HG is metabolised to α-KG by D-2HG dehydrogenase (D2HGDH). (**B**) qPCR confirming knockdown of *D2HGDH* by shRNA. (**C**) Migration of HCT116 cells with knockdown of *D2HGDH* and D-2HG treatment (250 μM) over 2 passages. (**D**) Invasion of HCT116 cells with knockdown of *D2HGDH* and D-2HG treatment (250 μM) over 2 passages. (**E**) *FN1* expression of HCT116 cells with knockdown of *D2HGDH* and D-2HG treatment (250 μM) over 2 passages. (**F**) *CDH1* expression of HCT116 cells with knockdown of D2HGDH and D-2HG treatment (250 μM) over 2 passages. Data are presented as means and standard deviations of at least three independent experiments; *p < 0.05, **p < 0.01.

Given that D-2HG can be metabolised to α-ketoglutarate by D2HGDH, it remained to be clarified whether the induction of EMT is a direct effect of D-2HG, or through its conversion to another metabolite (Fig. 4A). In order to resolve this ambiguity, D2HGDH was knocked down by RNA interference (Fig. 4B) to reduce the metabolisation of D-2HG and to enhance the direct effect of this metabolite. Knockdown of D2HGDH resulted in the enhancement of the induction of migration and invasion, in association with the increased expression of the mesenchymal marker in response to D-2HG octylester (Fig. 4C–E). Furthermore, knockdown of D2HGDH facilitated the reduction in the expression of the epithelial marker in response to D-2HG octylester (Fig. 4F). These findings suggest that D-2HG is directly responsible for the induction of EMT in colorectal cancer cells.

### D-2HG Induces EMT by Increasing the Expression of ZEB1 and Promotes the Trimethylation of Histone 3 Lysine 4 at the Promoter Region of the ZEB1 Gene

**Figure 5:**
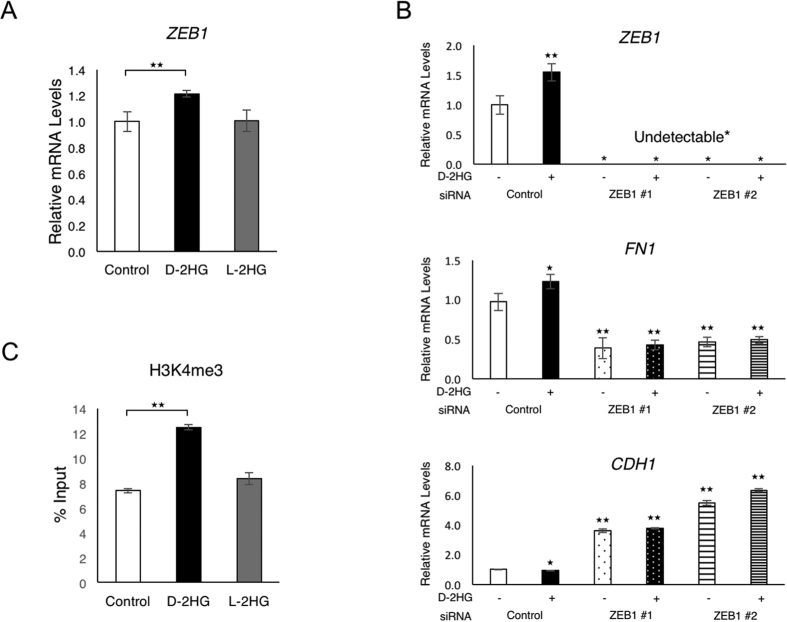
D-2HG increases the expression of *ZEB1* and the trimethylation of H3K4 at the promoter region of this gene. (**A**) qPCR of *ZEB1* in HCT116 cells treated by D- or L-2HG (250 μM) over 20 passages. (**B**) qPCR of *ZEB1*, *FN1* and *CDH1* in HCT116 cells treated by D-2HG (250 μM) and siRNA knockdown of *ZEB1*; *p < 0.05, **p < 0.01. (**C**) ChIP qPCR of trimethylated H3K4 at the promoter region of *ZEB1* in HCT116 cells treated by D- or L-2HG (250 μM) over 20 passages. Data are presented as means and standard deviations of at least three independent experiments; **p < 0.01.

The induction of EMT is orchestrated by transcription factors that switch on the expression of the mesenchymal genes and switch off the expression of the epithelial genes. Consistent with this, the administration of D-2HG octylester increased the expression of the *ZEB1* gene, which encodes one of the key transcription factors that regulate the induction of EMT (Fig. 5A). The expression of other transcription factors that regulate EMT did not increase in response to the administration of D-2HG octylester (Supplementary Fig. S3A). Furthermore, the induction of EMT by D-2HG was demonstrated to be dependent on increased ZEB1 expression, through the complete abrogation of the alteration in *CDH1* and *FN1* expression when siRNA knockdown of *ZEB1* was performed (Fig. 5B).

Previous reports highlight the alteration of gene expression by D-2HG through inhibition of Jumonji histone demethylase activity, resulting in the increased levels of histone methylation at the gene promoter regions^11^,^14^. ChIP-qPCR confirmed the increase in trimethylation of histone H3 lysine 4 (H3K4) at the promoter region of the *ZEB1* gene when D-2HG octylester was administered, which is a histone modification associated with increased gene expression (Fig. 5C). Alongside this, the acetylation of histone H3 lysine 9 (H3K9) and histone H3 lysine 27 (H3K27), both histone modifications associated with activation of gene expression, were also increased in the promoter region of the *ZEB1* gene in response to the administration of D-2HG octylester (Supplementary Fig. S3B). In contrast, there was no obvious change in the global levels of trimethylated H3K4 or acetylated histones (Supplementary Fig. S3C). These results signify the selective nature of histone modification by D-2HG, which in this case includes the promoter region of the *ZEB1* gene.

### D-2HG Level is Elevated in Colorectal Cancer, and Colorectal Cancer with Higher Levels of D-2HG are Associated with Distant Metastasis

**Figure 6:**
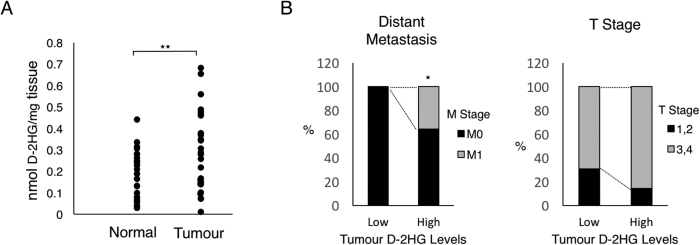
D-2HG is increased in colorectal cancer compared to non-cancerous tissue, and elevated D-2HG levels are associated with distant organ metastasis. (**A**) D-2HG were measured in fresh-frozen colorectal cancer and paired non-cancerous tissues (n = 28) by an enzymatic assay. (**B**) The colorectal cancer cases were divided according to whether the D-2HG levels were above or below the median value and assessed for frequencies of distant organ metastasis and T-stage. *p < 0.05, **p < 0.01.

In order to examine the clinical relevance of the above findings, D-2HG levels were measured in human colorectal cancer specimens (n = 28). This demonstrated D-2HG levels to be elevated in human colorectal cancer tissues compared to non-cancerous tissues, in the absence of mutations of *IDH1* or *IDH2* (Fig. 6A, Supplementary Table S2). Also when the colorectal cancer specimens were divided into two groups according to D-2HG levels at the median value, colorectal cancers with higher levels of D-2HG were associated with an increased frequency of distant metastasis, as well as an increased trend for a higher T-stage (Fig. 6B, Supplementary Table S2). These findings support the observations from *in vitro* and suggest the significant activity of D-2HG in inducing EMT and distant metastasis in human colorectal cancer.

## Discussion

In this study, we demonstrate that D- and L-2HG are elevated in some of the colorectal cancer cells, and that the D- enantiomer is directly responsible for inducing EMT through increased expression of *ZEB1*, a master regulator of EMT. Our data is consistent with the notion that D-2HG, an inhibitor of Jumonji-family histone demethylase^11^,^14^, induces EMT by increasing the trimethylation of H3K4 at the promoter region of the *ZEB1* gene, to increase the expression of this master regulator of EMT. We also found D-2HG to be elevated in colorectal cancer, particularly in the cases associated with distant metastasis, supporting a significant role of the oncometabolite in the human disease.

Previous metabolome analyses have shown that the concentrations of a number of metabolites to be deranged in malignancies^29^, including in colorectal cancer^30^. However, it has been challenging to demonstrate whether the altered metabolite levels are merely the consequence of the disease or whether they have an active role in driving the disease process. In this study, we provide further support that deranged metabolism drives the disease process in cancer, and that this is mediated by metabolites^8^,^31^. Our findings are also consistent with an earlier report that *IDH* mutation can induce EMT through the production of D-2HG and the increased expression of *ZEB1*^32^. Here we demonstrate the relevance and the significance of D-2HG in colorectal cancer, showing that endogenous D-2HG levels can be elevated by glutamine anaplerosis in the absence of *IDH* mutation, and that D-2HG levels are associated with distant metastasis.

The D-2HG concentrations measured in colorectal cancers were modest relative to cancers that harbour mutant *IDH*, such as leukaemias and brain tumours^11^,^33^,^34^,^35^. However our findings are consistent with the modest elevation of D-2HG reported in renal cell carcinoma, in which *IDH* mutation are also rare^22^. Importantly, colorectal cancers with higher D-2HG levels were more frequently associated with distant metastasis, consistent with the fact that even the modest increase in D-2HG is of clinical significance^24^.

Our study also highlights the discordant activity of the enantiomers D- and L-2HG in colorectal cancer, consistent with previous reports from other malignancies^12^,^13^. Despite L-2HG also being inhibitors of Tet2 and histone demethylases^36^, only the D- enantiomer of 2HG has been implicated in leukaemogenesis and astrocyte transformation^12^,^13^. This discordant activity has so far been explained by the ability of D-2HG, but not L-2HG, to stimulate the prolyl 4-hydroxylases activity of enzymes encoded by Egl nine homolog 1 (*EGLN1*) gene, and enhance the degradation of hypoxia inducible factor (HIF) transcription factor^12^,^13^. However, an earlier report of EMT being induced by HIF-1α in colorectal cancer^37^, suggested the induction of EMT by D-2HG does not occur through an axis involving HIF-1α. Indeed, the increase in the trimethylation of H3K4 being demonstrated at the promoter region of *ZEB1* in response to D-2HG provided an alternative explanation. Why the histone modification occurred only in response to D-2HG and not L-2HG is open to speculation, with one explanation being that there is a yet to be identified H3K4 demethylase that is inhibited only by D-2HG, and not L-2HG, active at the promoter regions of specific genes including *ZEB1*.

In addition to the trimethylation of H3K4, we also observed the acetylation of H3K9 and H3K27 at the promoter region of the *ZEB1* gene in response to D-2HG octylester. The acetylation of these lysine residues on histones is also associated with increased gene expression. The mechanism by which D-2HG causes histone acetylation remains to be elucidated, but may involve hitherto unreported interactions with histone acetyltransferases or histone deacetylases, or may be the knock-on effect of the H3K4 trimethylation, resulting in the recruitment of protein complexes that can alter the acetylation of neighbouring lysine residues. It would be worthwhile exploring the precise mechanisms by which D-2HG contributes to the disease process, given that epigenetic alterations can potentially be corrected in cancer, presenting with therapeutic opportunity^38^.

Reports from leukaemia demonstrate its dependence on elevated levels of D-2HG for oncogenesis and disease maintenance^12^,^39^, and early phase clinical trials of inhibitors against mutant IDH in leukaemia patients demonstrate provisional yet promising results^40^. The significance of D-2HG in colorectal cancer brought to light by the current study adds further weight to the importance of also investigating the role of IDH mutation occurring in a small subset of colorectal cancer. The same R132 IDH1 mutation that are found in leukaemia also occur in some cases of colorectal cancer^41^,^42^, but the significance of this IDH1 mutation in colorectal cancer is not yet known. We were limited in our study to using the DLD1 cell line that harbours an atypical mutation of IDH1 (G97D), because to the best of our knowledge of the literature there are no colorectal cancer cell lines that harbour the IDH1 mutation at R132, and justified in part by it being associated with high levels of 2HG nevertheless.

Cancer treatment targeting D-2HG has the potential to be both effective and safe, including for colorectal cancer. Further work is required to investigate the wider role of 2HG in colorectal cancer, including in oncogenesis. Interestingly, a recent report of 2HG being one of the very few metabolites whose levels are reduced in individuals administered aspirin^43^, currently the most effective drug available for chemoprevention of colorectal cancer^44^, tentatively suggests that D-2HG could be involved with oncogenesis of this disease. Furthermore, treatments targeting D-2HG can be envisaged to be safe, since there are no known physiological role of this metabolite^45^, which is normally maintained at low levels in health^46^.

In summary, we demonstrate D-2HG to be elevated in colorectal cancer and that it directly induces EMT in colorectal cancer cells, a phenotype associated with cellular invasion. The findings from human colorectal cancer specimens also support the significant role of D-2HG in promoting distant organ metastasis. Treatment strategies centred around reducing the levels of D-2HG or inhibiting its downstream effects in colorectal cancer could be effective.

## Experimental Procedures

### Cell Lines, Culture

The human colorectal cancer cell lines Caco2, DLD1, HCT116, HT29 and RKO were obtained from ATCC (VA) and the sarcoma cell line HT1080 was obtained from the Japan Cancer Research Resources Bank (Tokyo, Japan). The cell lines were cultured in Dulbecco’s modified Eagle’s medium (DMEM D6046; Sigma-Aldrich, MO), supplemented with 10% fetal bovine serum (Thermo Fisher Scientific, MA), and maintained at 37 °C and 5% CO_2_ in a humidified incubator. Cells were reseeded every 3–4 days, prior to reaching confluency.

### Reagents and Antibodies

2HG octylesters were purchased as separate enantiomers, D-2HG octylester (16366, Cayman Chemical Company, MI) or L-2HG octylester (16367, Cayman Chemical Company, MI). The antibodies used for Western blot analysis were glutaminase antibody (ab93434, Abcam, 1:000 dilution) and E-cadherin antibody (#3195, Cell Signaling Technology, 1:1000 dilution).

### Cell Proliferation Assay

Cells were seeded at 1,000 cells per well in 96-well plates with 200 μL of culture medium. At the indicated time points, the cells were fixed with 25% glutaraldehyde and stained with 0.05% crystal violet/20% methanol solution. After washing away the excess crystal violet, 0.05 M NaH_2_PO_4_/50% Et-OH was added and colometric quantification was conducted with the absorbance set at 595 nm.

### Small RNA Interference and Transfection

Small interfering RNA (siRNA) targeting GLS1 (NM_001256310.1, NM_014905.4) or ZEB1 (NM_001128128.2) was used, alongside nonspecific siRNA as negative control (Thermo Fisher Scientific, MA). At 50% confluency, the siRNA was transfected with Lipofectamine RNA iMax (Thermo Fisher Scientific, MA).

### shRNA Construct and Lentivirus Production

Lentiviral shRNA construct, TRCN0000064034 targeting human *D2HGDH* was obtained from the MISSION TRC-Hs1.0 library (Sigma-Aldrich, MO). Control cells were transfected using the same procedure but with an empty control vector. HCT116 cells were selected using 1 μg/ml of puromycin over 2 weeks to establish stable cell lines.

### Quantitative PCR

Total RNA was extracted from cultured cells or clinical specimens using phenol followed by precipitation (TRIzol kit, Thermo Fisher Scientific, MA). cDNA was synthesized by reverse transcription using ReverTra Ace (TOYOBO, Osaka, Japan). Quantitative PCRs was performed with the LightCycler (Roche Applied Science, Penzberg, Germany) and Thunderbird (TOYOBO, Osaka, Japan). The primer sequences used in this study are summarized in Supplementary Table S3.

### Sanger Sequencing

Genomic DNA was extracted from cultured cells using a commercially available kit (QIAGEN, Hilden, Germany). Sequencing of IDH1 used primers covering amino acid residues 41–138, and sequencing of IDH2 used primers covering amino acid residues 125–226. The coding region and the intron/exon boundaries of *D2HGDH* and *L2HGDH* were also sequenced. The primers used for sequencing are detailed in Supplementary Table S4. Cycle sequencing was carried out using the BigDye Terminator v3.1 Cycle Sequencing kit (Thermo Fisher Scientific, MA).

### Western Blot

After washing the cells in ice-cold phosphate buffer solution (PBS), cells were pelleted and total protein extracts were made by lysing in radio-immunoprecipitation assay (RIPA) buffer (Thermo Fisher Scientific, MA). Cell lysates were measured for their protein concentrations using the Bradford method (BioRad, CA), after which they were separated by sodium dodecyl sulfate–polyacrylamide gel electrophoresis, then transferred to polyvinylidene difluoride membranes using iBlot (Life Technologies, CA). The membranes were incubated in primary antibodies overnight at 4 °C, then incubated in HRP-linked secondary antibodies (GE Healthcare Life Sciences, Little Chalfont, UK) for 1 hour at room temperature. Antigen-antibody complexes were visualized with chemiluminescence (BioRad, CA).

### Flow Cytometry

E-cadherin expression was determined by flow cytometry. Cells were detached with versene and suspended in ice cold PBS with 5 μL E-cadherin-AlexaFluor 488 (#324110, BioLegend). After 1 hour of incubation, each sample was analysed using the BD FACSAria^TM^ II instrument (BD Bioscience).

### Wound Healing Assay

Cells were seeded in 6 well plates in triplicates in numbers sufficient to become confluent within 1–2 days. A wound was then introduced using the tip of a P200 pipette, after which the cells were washed in PBS and cultured in DMEM with 1% FBS. The width of the wounds was assessed at the indicated time points, taking the mean of the narrowest and the widest points in a microscopic field.

### Invasion Assay

5 × 10^4^ HCT116 cells were seeded into the upper chamber of the 24-well BioCoat Matrigel Invasion Assay (BD Biosciences, San Jose) in triplicates in DMEM without FBS. DMEM containing 10% FBS was added to the lower chamber to induce cell invasion by chemotaxis. After 48 hours, the non-invaded cells remaining above the membrane were removed with cotton swabs, whilst the cells that had invaded through the membrane were stained using the Diff Quik kit (Sysmex, Kobe, Japan). The number of cells that had invaded through the matrigel in three random microscopic fields were counted in each chamber.

### Gas Chromatography-Mass Spectrometry

Derivatisation was conducted in order to measure D- and L-2HG separately as (D and L)-0-acetyl-2-HG acid di-(D)-2-butyl esters, using GC-MS as previously described^47^. Disodium D-2HG (H8378, Sigma-Aldrich), Disodium L-2HG (90790, Sigma-Aldrich), and Disodium(RS)-2-hydroxy-1,5-pentanedioate-2,3,3-d3 CDN, (D-7496, C/D/N Isotopes, Pointe-Claire, Canada) were used as standards and for final quantification of the metabolites. Briefly, cell pellets stored at −80 °C were lysed in water and acidified to pH1-2 with HCl. After extraction with ethyl acetate and drying under nitrogen, tri-TMS derivatives of 2HG were made, before butylation and then acetylation. After evaporation and dissolving the extracts in chloroform, samples were injected to an Agilent 7890A GC equipped with an Agilent 5975C mass selective detector operating in splitless mode, using electron impact ionization at ionizing voltage of 70 eV and electron multiplier set to 1060 V. Helium was used for chamber gas at a flow rate of 30 cm/sec. GC temperature started at 120 °C for 3 minutes, ramped to 230 °C at 4 °C/min and held for 5 minutes, then ramped to 300 °C at 20 °C/min and held for 10 minutes. Mass range of 100–600 m/z was recorded at 2.71 scans/second.

### Enzymatic Assay for D-2HG

The enzymatic assay for D-2HG (K213-100, BioVision, CA) was used according to the manufacturer’s instructions. Briefly, 10–20 mg of tissues stored at −80 °C were homogenized in the supplied buffer solution using the TaKaRa BioMasher (9791A, Clontech Laboratories, CA). The sample solution was deproteinised using a kit (K808-200, BioVision, CA) according to the manufacturer’s protocol, mixed with D-2HG substrate mix and D2HGDH enzyme and incubated at 37 °C for 1 hour, after which colometric assessment was made at OD_450nm_.

### Clinical Tissue Samples

Colorectal cancer specimens were harvested at the time of surgical resection in the Department of Gastroenterological Surgery, Osaka University during 2011. Written informed consent was obtained from all patients regarding the use of the resected specimens in this study, and all experimental methods involving human tissues were carried out in accordance with the guidelines and regulations approved by the Ethics Committee at the Graduate School of Medicine, Osaka University (Approval Number 15222-2). None of the patients underwent neoadjuvant chemo- or radiotherapy. Samples were immediately frozen at −80 °C for storage. The specimens were analysed under the approval of the Ethics Committee at the Graduate School of Medicine, Osaka University (Approval Number 15222-2).

### Statistical analysis

Data is presented as mean ± standard deviation. Students’ t-test and Fisher’s exact test were used where appropriate to determine whether differences were statistically significant using JMP Pro 12 software (SAS Institute, Cary, NC). *P* < 0.05 was considered to be statistically significant.

## Additional Information

**How to cite this article**: Colvin, H. *et al*. Oncometabolite D-2-Hydroxyglurate Directly Induces Epithelial-Mesenchymal Transition and is Associated with Distant Metastasis in Colorectal Cancer. *Sci. Rep.* **6**, 36289; doi: 10.1038/srep36289 (2016).

**Publisher’s note:** Springer Nature remains neutral with regard to jurisdictional claims in published maps and institutional affiliations.

## Supplementary Material

Supplementary Information
