# Supplementary material for: Oncometabolite D-2-Hydroxyglurate Directly Induces Epithelial-Mesenchymal Transition and is Associated with Distant Metastasis in Colorectal Cancer
Source: Sci Rep. 2016 Nov 8;6:36289. doi: 10.1038/srep36289 (PMC5099755; doi:10.1038/srep36289)
Supplement: Supplementary Information [file srep36289-s1.doc]

**Supplementary Information**

**Oncometabolite D-2-Hydroxyglurate Directly Induces Epithelial-Mesenchymal Transition and is Associated with Distant Metastasis in Colorectal Cancer**

**Authors:** Hugh Colvin^1,2^,*, Naohiro Nishida^2^,*, Masamitsu Konno^2^, Naotsugu Haraguchi^1^, Hidekazu Takahashi^1^, Junichi Nishimura^1^, Taishi Hata^1^, Koichi Kawamoto^1,2^, Ayumu Asai^3^, Kenta Tsunekuni^1,2,4^, Jun Koseki^3^, Tsunekazu Mizushima^1^, Taroh Satoh^2^, , Yuichiro Doki^1,2,3^, Masaki Mori^1,2,3^, Hideshi Ishii^2,3^

**Affiliations:** 1) Department of Gastrointestinal Surgery, Osaka University Graduate School of Medicine, Osaka, 565-0871, Japan; 2) Department of Frontier Science for Cancer and Chemotherapy, Osaka University Graduate School of Medicine, Osaka, 565-0871, Japan; 3) Department of Cancer Profiling Discovery, Osaka University Graduate School of Medicine, Osaka, 565-0871, Japan; 4) Taiho Pharmaceutical Co., Ltd., Chiyoda-ku, Tokyo, 101-0054, Japan

*****H.C. and N.N. contributed equally to this work.

Correspondence and requests for materials should be addressed to M.M. or H.I.

**Supplementary Figure S1**

**
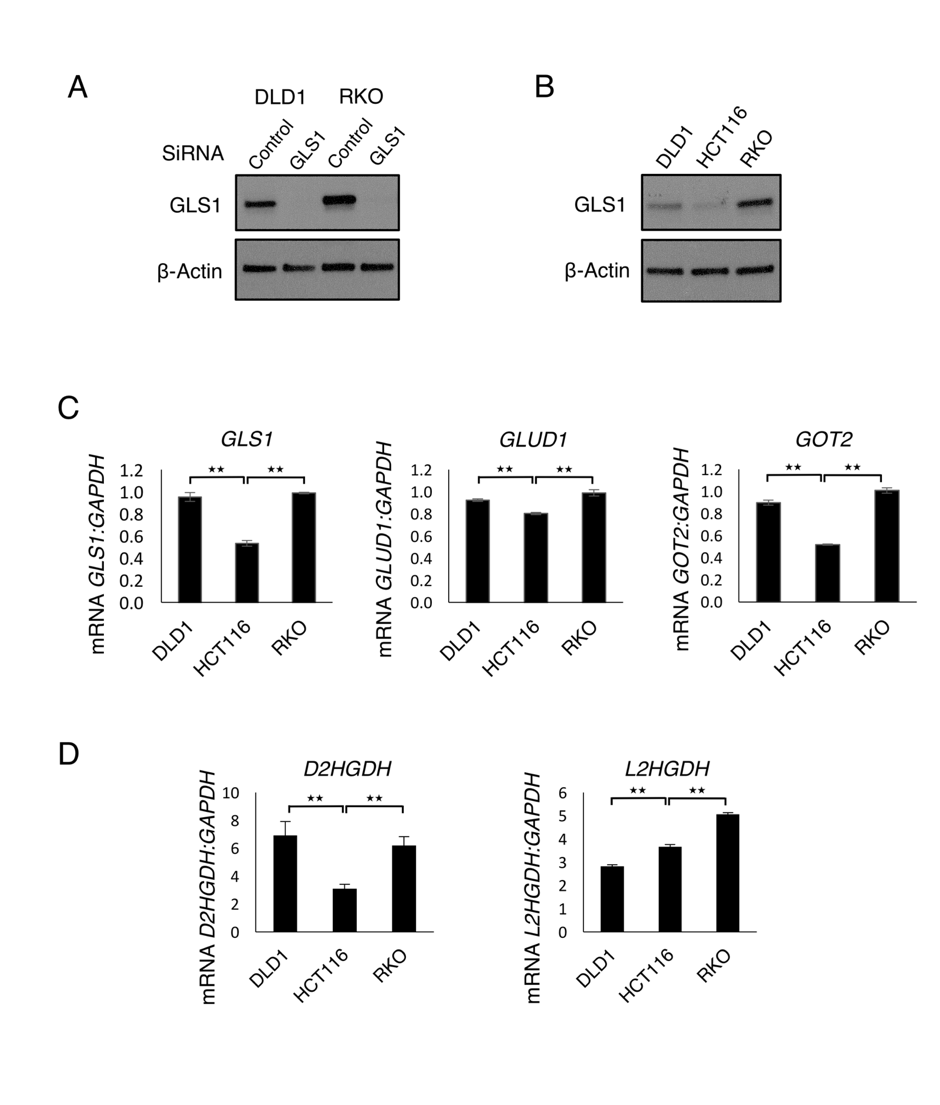
**

**Supplementary Figure S2**

**
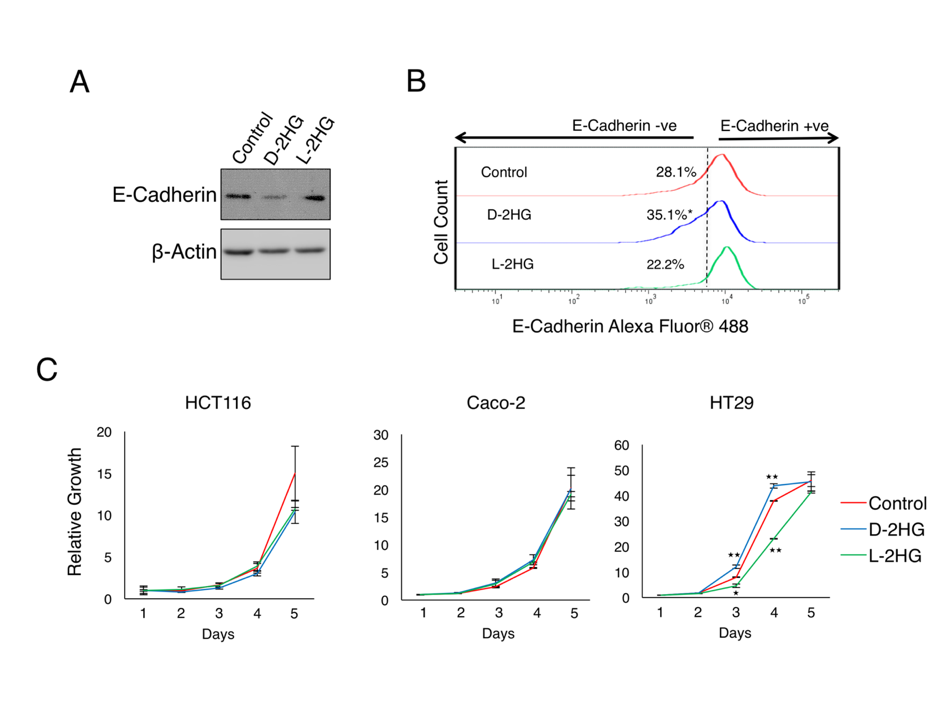
**

**Supplementary Figure S3**

**
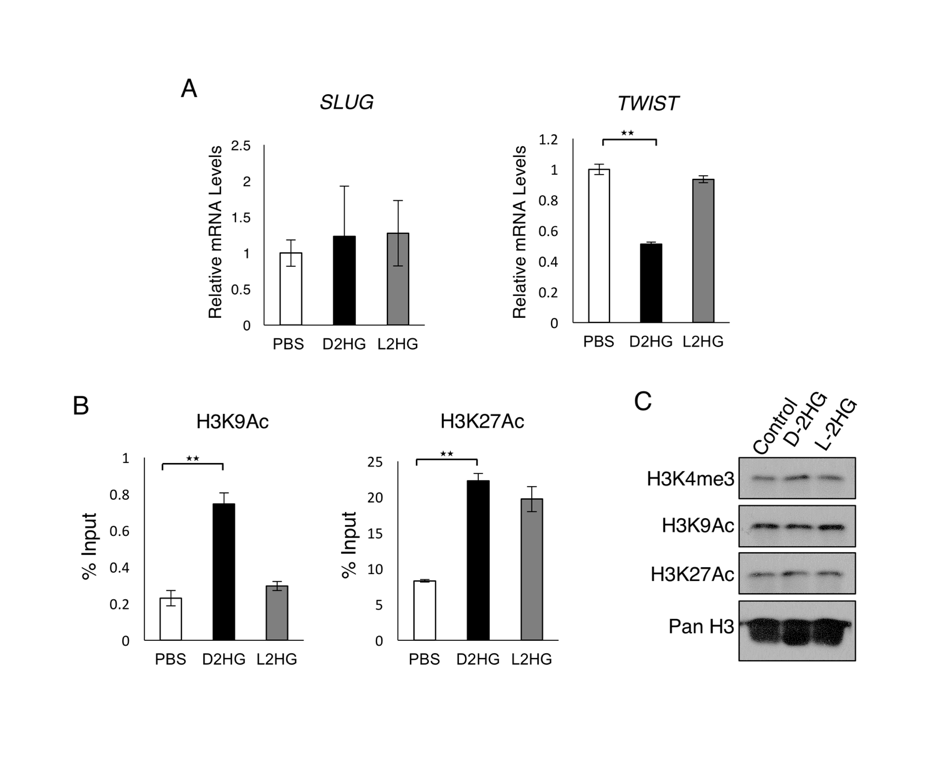
**

**Supplementary Table 1**

| **Mutations** | **HCT116** | **HT29** | **Caco2** |
| --- | --- | --- | --- |
| **MSI Status** | MSI | MSS | MSS |
| **CIMP Panel 1** | + | + | + |
| **CIMP Panel 2** | + | + | - |
| **CIN** | - | + | + |
| ***KRAS*** | G13D | WT | WT |
| ***BRAF*** | WT | V600E | WT |
| ***PIK3CA*** | H1047R | P449T | WT |
| ***PTEN*** | WT | WT | WT |
| ***TP53*** | WT | R273H | E204X |

**Supplementary Table 3**

| **Clinicopathological Factors** | **Classification** | **N** | **D-2HG Low** | **D-2HG High** | **P** |
| --- | --- | --- | --- | --- | --- |
| **Patient Background** | | | | | |
| Sex | Male | 20 | 10 | 10 | 1.0000 |
|  | Female | 8 | 4 | 4 |  |
| Age | <65 | 14 | 5 | 9 | 0.2568 |
|  | ≥65 | 14 | 9 | 5 |  |
| *IDH1* or *2* Mutations | Present | 0 | 0 | 0 | 1.0000 |
|  | Absent | 28 | 14 | 14 |  |
| **Tumour Characteristics** | | | | | |
| Histological type | Tub1, Tub2, Pap | 26 | 13 | 13 | 1.0000 |
|  | Poor, Muc | 2 | 1 | 1 |  |
| Depth of tumor invasion | T1, T2 | 5 | 4 | 1 | 0.3259 |
|  | T3, T4 | 23 | 10 | 13 |  |
| Lymph node metastasis | Positive | 14 | 7 | 7 | 1.0000 |
|  | Negative | 14 | 7 | 7 |  |
| Distant metastasis | Positive | 5 | 0 | 5 | 0.0407 |
|  | Negative | 23 | 14 | 9 |  |
| Lymphatic invasion | Positive | 23 | 11 | 12 | 1.0000 |
|  | Negative | 5 | 3 | 2 |  |
| Venous invasion | Positive | 15 | 6 | 9 | 0.4495 |
|  | Negative | 13 | 8 | 5 |  |
| Stage | Ⅰ, Ⅱ | 13 | 7 | 6 | 1.0000 |
|  | Ⅲ, Ⅳ | 15 | 7 | 8 |  |

**Supplementary Table 3**

| **Genes** | **Primer Sequences** |
| --- | --- |
| ***CDH1*** | Forward: 5’-ACACCATCCTCAGCCAAGA-3’  Reverse: 5’-CGTAGGGAAACTCTCTCGGT-3’ |
| ***D2HGDH*** | Forward: 5’-CACGGTGTCCATCTTGTGTC-3’  Reverse: 5’-TCTGCAGAACCTCAGCAAAG-3’ |
| ***FN1*** | Forward: 5’-CTGGCCGAAAATACATTGTAAA-3’  Reverse: 5’-CCACAGTCGGGTCAGGAG-3’ |
| ***GAPDH*** | Forward: 5’-AACGGGAAGCTTGTCATCAATGGAAA-3’  Reverse: 5’-GCATCAGCAGAGGGGGCAGAG-3’ |
| ***GLS1*** | Forward: 5’-TGGTGGCCTCAGGTGAAAAT-3’  Reverse: 5’-CCAAGCTAGGTAACAGACCCTGTTT-3’ |
| ***GLUD1*** | Forward: 5’-GGTCATCGAAGGCTACCG-3’  Reverse: 5’-TCAGTGCTGTAACGGATACCTC-3’ |
| ***GOT2*** | Forward: 5’-GACCAAATTGGCATGTTCTGT-3’  Reverse: 5’-CGGCCATCTTTTGTCATGTA-3’ |
| ***L2HGDH*** | Forward: 5’-TCAAAAATTCATCCCTGAAATTACT-3’  Reverse: 5’-CTACCAGATTTCCATCTCTATCCAG-3’ |
| ***SLUG*** | Forward: 5’-TCAGCTCAGGAGCATACAGC-3’  Reverse: 5’-GACTCACTCGCCCCAAAGA-3’ |
| ***SNAI1*** | Forward: 5’-GCTGCAGGACTCTAATCCAGA-3’  Reverse: 5’-ATCTCCGGAGGTGGGATG-3’ |
| ***TWIST*** | Forward: 5’-CGGACAAGCTGAGCAAGATT-3’  Reverse: 5’-CCTTCTCTGGAAACAATGAC-3’ |
| ***VIM*** | Forward: 5’-TCCAGCAGCTTCCTGTAGGT-3’  Reverse: 5’-CCCTCACCTGTGAAGTGGAT-3’ |
| ***ZEB1*** | Forward: 5’-AACTGCTGGGAGGATGACAC-3’  Reverse: 5’-TCCTGCTTCATCTGCCTGA-3’ |
| ***SNAI1* ChIP qPCR** | Forward: 5’-CTACAGCGAGCTGCAGGACTCTAAT-3’  Reverse: 5’-ACAAAACATCCTGTGACTCGATCC-3’ |
| ***ZEB1* ChIP qPCR** | Forward: 5’-CAGTTTGGAGAGACGTTGTAAG-3’  Reverse: 5’-CTCTCGCCACAGGAACTGTC-3’ |

**Supplementary Table 4**

| **Genes** | **Primer Sequences** |
| --- | --- |
| ***D2HGDH* Exon 2** | Forward: 5’-TGCTTCTGCAAGCGTGTTTC-3’  Reverse: 5’-TTTGAAGCCTCCACGGGAAG-3’ |
| ***D2HGDH* Exon 3** | Forward: 5’-GAGTGACCACTTGCCTCATC-3’  Reverse: 5’-AACCAAGATGTCATCGGCTG-3’ |
| ***D2HGDH* Exon 4** | Forward: 5’- GCAGGGTAATCAGGATTTGG -3’  Reverse: 5’- GCCCTAACTCATTCACCCAC -3’ |
| ***D2HGDH* Exon 5** | Forward: 5’- GTTCCTTCTGGGTGGCTTG -3’  Reverse: 5’- ATGAGAGCCGTGAGAGGAC -3’ |
| ***D2HGDH* Exon 6** | Forward: 5’- GTCCATCCTTCAGCCTCTTG -3’  Reverse: 5’- CTTCCTCACACCAACAGTG -3’ |
| ***D2HGDH* Exon 7** | Forward: 5’- TGTTTGTTGCAGTGCCAGTC -3’  Reverse: 5’- TGTGTCCAGACGTGCAGAAG -3’ |
| ***D2HGDH* Exon 8** | Forward: 5’- TCTTGGCCACGAAAGATCAG -3’  Reverse: 5’- CTGTCTAGGCTGCACCAATG -3’ |
| ***D2HGDH* Exon 9** | Forward: 5’- ATACAGAACATGCTGCTGCC -3’  Reverse: 5’- GATATGCTAAGCCAGAGACC -3’ |
| ***D2HGDH* Exon 10** | Forward: 5’- ATCTTGGGAGGGGCTGTTG -3’  Reverse: 5’- TTGGCAGCAGCAGGAGTG -3’ |
| ***L2HGDH* Exon 1** | Forward: 5’‐AAGGCGCGCCACTTCATTG‐3’  Reverse: 5’‐CGGGACAGGGAAATACGAAC‐3’ |
| ***L2HGDH* Exon 2** | Forward: 5’‐TGCATGTGAAGTTTGGCGAG‐3’  Reverse: 5’‐CACTGACATTCAGCATGAAAG‐3’ |
| ***L2HGDH* Exon 3** | Forward: 5’‐ACATAACGTCACACCATCTTTTT‐3’  Reverse: 5’‐GGCAGAATTTTACATGATGTGG‐3’ |
| ***L2HGDH* Exon 4** | Forward: 5’‐CTCCTTTGGGTCATACAATAG‐3’  Reverse: 5’‐CTGTGACAGGATTATCTAACTG‐3’ |
| ***L2HGDH* Exon 5** | Forward: 5’‐TAGCAGCAAGAAAAGCTTGG‐3’  Reverse: 5’‐ATGGAGGGCTGACTATATTC‐3’ |
| ***L2HGDH* Exon 6** | Forward: 5’‐GGTGCAATCATAGTAATGAC‐3’  Reverse: 5’‐ACTTAAAATACAGCCCTGTG‐3’ |
| ***L2HGDH* Exon 7** | Forward: 5’‐CCCTCTTGACCTATTCTAC‐3’  Reverse: 5’‐CATCTCCTTTATGACCACC‐3’ |
| ***L2HGDH* Exon 8** | Forward: 5’‐TGCATGAGAAGAAAGTGTTTTATG‐3’  Reverse: 5’‐CCAATCACAAATATGGGGATTTAC‐3’ |
| ***L2HGDH* Exon 9** | Forward: 5’‐GCCTAGATTTTTGTGATGAC ‐3’  Reverse: 5’‐GTATTTACACTCCTTATCCC ‐3’ |
| ***L2HGDH* Exon 10** | Forward: 5’‐CGCTGACTTGTAAAGTATCC‐3’  Reverse: 5’‐TGCAGTGGTTATCTTTGACC‐3’ |
| ***IDH1***  **(Amino Acid Residue 41-138)** | Forward: 5’-TGTGTTGAGATGGACGCCTA-3’  Reverse: 5’-GGTGTACTCAGAGCCTTCGC-3’ |
| ***IDH2***  **(Amino Acid Residue 125-226)** | Forward: 5’-CTGCCTCTTTGTGG CCTAAG-3’  Reverse: 5’-ATTCTGGTTGAAAGATGGCG-3’ |

**Supplementary Figure Legends**

**Supplementary Figure S1. D- and L-2HG levels are elevated in colorectal cancer cells and is dependent on glutamine metabolism.** (A) Western blot confirming the knockdown of GLS1 by siRNA. (B) Western blot of the basal expression of GLS1 in colorectal cancer cell lines (DLD1, HCT116, RKO). (C) qPCR of the basal expressions of *GLS1*, glutamate dehydrogenase 1 (*GLUD1*) and aspartate aminotransferase 2 (*GOT2*) in the 3 colorectal cancer cell lines. Data are presented as means and standard deviations of at least three independent experiments; ^★★^*p* < 0.01. (D) qPCR of the basal expressions of *D2HGDH* and *L2HGDH* in the 3 colorectal cancer cell lines. Data are presented as means and standard deviations of at least three independent experiments; ^★★^*p* < 0.01.

**Supplementary Figure S2. D-2HG induces epithelial-mesenchymal transition in colorectal cancer cells.** (A) Western blot of epithelial marker E-cadherin (*CDH1*) in HCT116 cells treated with D- or L-2HG (250μM) over 20 passages. (B) FACS analysis of epithelial marker E-cadherin (*CDH1*) in HCT116 cells treated with D- or L-2HG (250μM) over 20 passages; *p* < 0.05. (C) Proliferation assay of colorectal cancer cell lines treated with D- or L-2HG (250μM), over 20 passages (HCT116), 4 passages (Caco-2) or 2 passages (HT29). Data are presented as means and standard deviations of at least three independent experiments; ^★^*p* < 0.05, ^★★^*p* < 0.01.

**Supplementary Figure S3. D-2HG increases the expression of *ZEB1* and the trimethylation of H3K4 at the promoter region of this gene.** (A) qPCR of *SLUG* and *TWIST* in HCT116 cells treated with D- or L-2HG (250μM) over 20 passages. (B) ChIP qPCR of acetylated H3K9 or H3K27 at the promoter region of *ZEB1*. (C) Western blot representing the global levels of trimethylated H3K4, acetylated H3K9, and acetylated H3K27 in HCT116 cells treated with D- or L-2HG (250μM) over 20 passages. Data are presented as means and standard deviations of at least three independent experiments; ^★★^*p* < 0.01.

**Supplementary Table Legends**

**Supplementary Table 1. The genetic background of the colorectal cancer cell lines.** Abbreviations; MSI=microsatellite instability, MSS=microsatellite stable, CIMP= CpG island methylator phenotype, CIN=chromosomal instability pathway, WT=wild type. Information taken from Forbes S. A. et al. COSMIC: Exploring the world’s knowledge of somatic mutations in human cancer. Nucleic Acids Res. 43, D805–D811 (2015).

**Supplementary Table 2. Clinicopathological features of colorectal cancer specimens according to D-2HG levels.** The colorectal cancer specimens were divided into low and high groups depending on their D-2HG levels relative to the median value.

**Supplementary Table 3. The list of primers used for PCR.**

**Supplementary Table 4. The list of primers used for PCR and sequencing.**
